# Phosphoglucomutase 1 contributes to optimal cyst development in *Toxoplasma gondii*

**DOI:** 10.1186/s13104-022-06073-5

**Published:** 2022-05-21

**Authors:** Emily V. Quach, Binh Cao, Edres Babacarkhial, Daniel Ho, Janak Sharma, Pascale S. Guiton

**Affiliations:** 1 Department of Biology, Laney College, Oakland, CA USA; 2 School of Medicine, University of California San Francisco, San Francisco, CA USA; 3 Department of Biological Sciences, California State University East Bay, Hayward, CA USA; 4 Department of Pathology, Stanford University School of Medicine, Stanford, CA USA

**Keywords:** Phosphoglucomutase, Glycolysis, Gluconeogenesis, *Toxoplasma*, Amylopectin, Tissue cysts, Stage conversion

## Abstract

**Objective:**

*Toxoplasma gondii* is a ubiquitous parasite of medical and veterinary importance; however, there exists no cure for chronic toxoplasmosis. Metabolic enzymes required for the production and maintenance of tissue cysts represent promising targets for novel therapies. Here, we use reverse genetics to investigate the role of *Toxoplasma* phosphoglucomutase 1, PGM1, in *Toxoplasma* growth and cystogenesis.

**Results:**

We found that disruption of *pgm1* did not significantly affect *Toxoplasma* intracellular growth and the lytic cycle. *pgm1-*defective parasites could differentiate into bradyzoites and produced cysts containing amylopectin in vitro. However, cysts produced in the absence of *pgm1* were significantly smaller than wildtype. Together, our findings suggest that PGM1 is dispensable for in vitro growth but contributes to optimal *Toxoplasma* cyst development in vitro, thereby necessitating further investigation into the function of this enzyme in *Toxoplasma* persistence in its host.

**Supplementary Information:**

The online version contains supplementary material available at 10.1186/s13104-022-06073-5.

## Introduction

*Toxoplasma gondii* is an obligate intracellular protozoan responsible for toxoplasmosis in humans and other warm-blooded animals. Infections occur mostly from consuming contaminated water, food, or undercooked meat from chronically infected animals [1]. Bradyzoites inside tissue cysts are released into the gastrointestinal tract where they invade enterocytes and convert to tachyzoites inside a parasitophorous vacuole (PV). Tachyzoites replicate rapidly, eventually lysing out of the host cell to disseminate throughout the body. In response to stressful stimuli, they convert back to bradyzoites which remain encysted in the brain and skeletal muscles for life [2]. Chronic toxoplasmosis is incurable and parasite reactivation life-threatening, particularly for the immunocompromised [3].

Bradyzoites are replete with cytoplasmic amylopectin granules [4]. The tight regulation of enzymes involved in metabolizing this polysaccharide is critical for tissue cyst production and survival during chronic infection [5–8]. Phosphoglucomutases (PGMs) catalyze the interconversion of glucose-1-phosphate to glucose-6-phosphate [9], effectively linking amylopectin metabolism to glycolysis in this parasite. Both PGM paralogs in *Toxoplasma* [10], PGM1, also known as parafusin-related *Toxoplasma* protein 1 (PRP1) [11], and PGM2, are upregulated during chronic infection in mice [12] and have been implicated in calcium (Ca^2+^)-dependent signaling for microneme secretion [13–15].

Here, we used the CRISPR/Cas9 gene-editing system [16] to disrupt *pgm1* in a cyst-forming *Toxoplasma* strain. Our data show that this mutation did not prevent intracellular replication or the completion of the lytic cycle. While both strains could produce amylopectin-containing cysts, we found that *pgm1*-defective cysts are significantly smaller than the parental cysts. Together, our findings corroborate previous reports that PGM1 is dispensable for *Toxoplasma* viability and demonstrate that the enzyme contributes to optimal cyst development in vitro.

## Main text

### Materials and methods

#### Parasite and host cells

Human foreskin fibroblasts (HFFs) and Me49Δ*hxgprt*, a Type II strain of *Toxoplasma* lacking hypoxanthine-xanthine-guanine phosphoribosyltransferase (HXGPRT), were kind gifts from John Boothroyd at Stanford University. Parasites were maintained in HFFs in Dulbecco’s modified Eagle medium supplemented with 10% fetal bovine serum, 2 mM L-glutamine, 2.5 µg/ml fungizone, 100 U/ml penicillin, and 100 µg/ml streptomycin (cDMEM) at 37 ºC and 5% CO_2_.

### Disruption of *pgm1*

All primers used in this study are listed in Additional file 1. pSAG::Cas9-U6::sgPGM1 was obtained by substitution of sgUPRT with sgPGM1 in pSAG1::Cas9-U6::sgUPRT [16] using Q5 site-directed mutagenesis (New England Biolabs Inc, NEB). pUC19 modified to express *hxgprt* under the dihydrofolate reductase (DHFR) promoter using standard molecular cloning techniques to create pDHFR::*hxgprt*. Freshly released Me49Δ*hxgprt* (WT) were transfected with pSAG1::Cas9-U6::sgUPRT and linearized pDHFR::*hxgprt* at a 1:3 molar ratio in a 4 mm gap cuvette in an BTX ECM 630 Exponential Decay Wave electroporator system (BTX Harvard Apparatus) [16]. Transgenic Me49Δ*hxgprt*Δ*pgm1* parasites (Δ*pgm1)* were obtained after 10 days of selection in cDMEM containing 25 µg/ml of mycophenolic acid and 50 µg/ml xanthine and cloned by limiting dilutions [17]. Disruption of *pgm1* and integration of the selection cassette were confirmed by polymerase chain reaction (PCR) and DNA sequencing (Elim Biopharmaceuticals Inc).

### Replication assay

Freshly released parasites were centrifuged at 1500 rpm for 10 min and washed once with 1XPBS. Confluent HFFs on glass coverslips were infected with 1.2 × 10^5^ parasites in cDMEM for 24 h. The number of parasites per vacuole was determined by immunofluorescence microscopy, as previously described [18], following staining with mouse α-SAG1 and rabbit α-GRA7 obtained from the Boothroyd lab. Immunostaining and visualization are further described below.

### Plaque assay

WT and Δ*pgm1* tachyzoites were syringe-lysed through a 27G needle and passed through a 5 µm filter. Confluent HFFs were infected with 250 parasites in cDMEM and incubated at 37 ºC with 5% CO_2_ for 10 days undisturbed. Following methanol fixation and crystal violet staining, plaque numbers and sizes were determined using a stereoscope (Leica EZ4) and ImageJ version 1.52A (National Institutes of Health) [19, 20].

### Tachyzoite-to-bradyzoite differentiation

Tachyzoites were induced to differentiate into bradyzoites in HFFs as previously described [21]. Briefly, confluent HFFs on glass coverslips were infected with 4.8 × 10^4^ parasites for 3 h in cDMEM before replacing the medium with Switch Medium (RPMI 1640 supplemented with 1% fetal bovine serum, 100 U/ml penicillin, and 100 µg/ml streptomycin, 10 mg/mL HEPES, pH 8.2). Parasites were incubated for 4 days at 37 ºC with ambient CO_2_ and the medium was changed every 24 h to maintain alkaline conditions.

### Immunostaining fluorescence assay and amylopectin staining

Infected monolayers were washed with phosphate-buffered saline (PBS) and fixed with 4% paraformaldehyde (Electron Microscopy Sciences) for 15 min at room temperature (RT). Cells were permeabilized with 0.2% or 0.4% Triton X-100 for 20 min and incubated for 1 h in 3% Bovine Serum Albumin (BSA; Fisher Scientific) in PBS. Primary antibodies diluted in 3% BSA/PBS (mouse α-SAG1 1:10,000, rabbit α-GRA7 1:1000) were added to the monolayers, when indicated, and incubated overnight at 4 ºC. Unbound antibodies were washed away with three 5 min washes in 1XPBS. The cells were then stained with secondary antibodies in 3% BSA/PBS (Goat α -Mouse 546 or Goat α-Rabbit 488 at 1:5000) for 45 min at RT. *Dolichos biflorus* Agglutinin (DBA; Vector Laboratories) was used at 1:100 to detect the cyst wall. After washing as described above, the coverslips were mounted with VECTASHIELD Mounting Medium containing DAPI (Vector Laboratories). Amylopectin was stained with Periodic Acid Schiff (PAS; Fisher Scientific) according to the manufacturer’s guidelines.

Immunofluorescence images were obtained using an inverted microscope (Leica DM IL LED) with 100 × oil immersion objective. The number of parasites (SAG1-positive) inside individual vacuole (GRA7 +) from randomly selected fields was determined from direct count under the microscope. The areas of plaques and cysts, both selected from random fields of view, were determined using ImageJ version 1.52A and 1.53, respectively [19, 20].

### Statistical methods

Statistical analyses were performed using GraphPad Prism version 8.4.3. A *p*-value ≤ 0.05 was considered a statistically significant difference between groups.

## Results

### *Toxoplasma* phosphoglucomutases are upregulated during chronic infection in mice

**Fig. 1:**
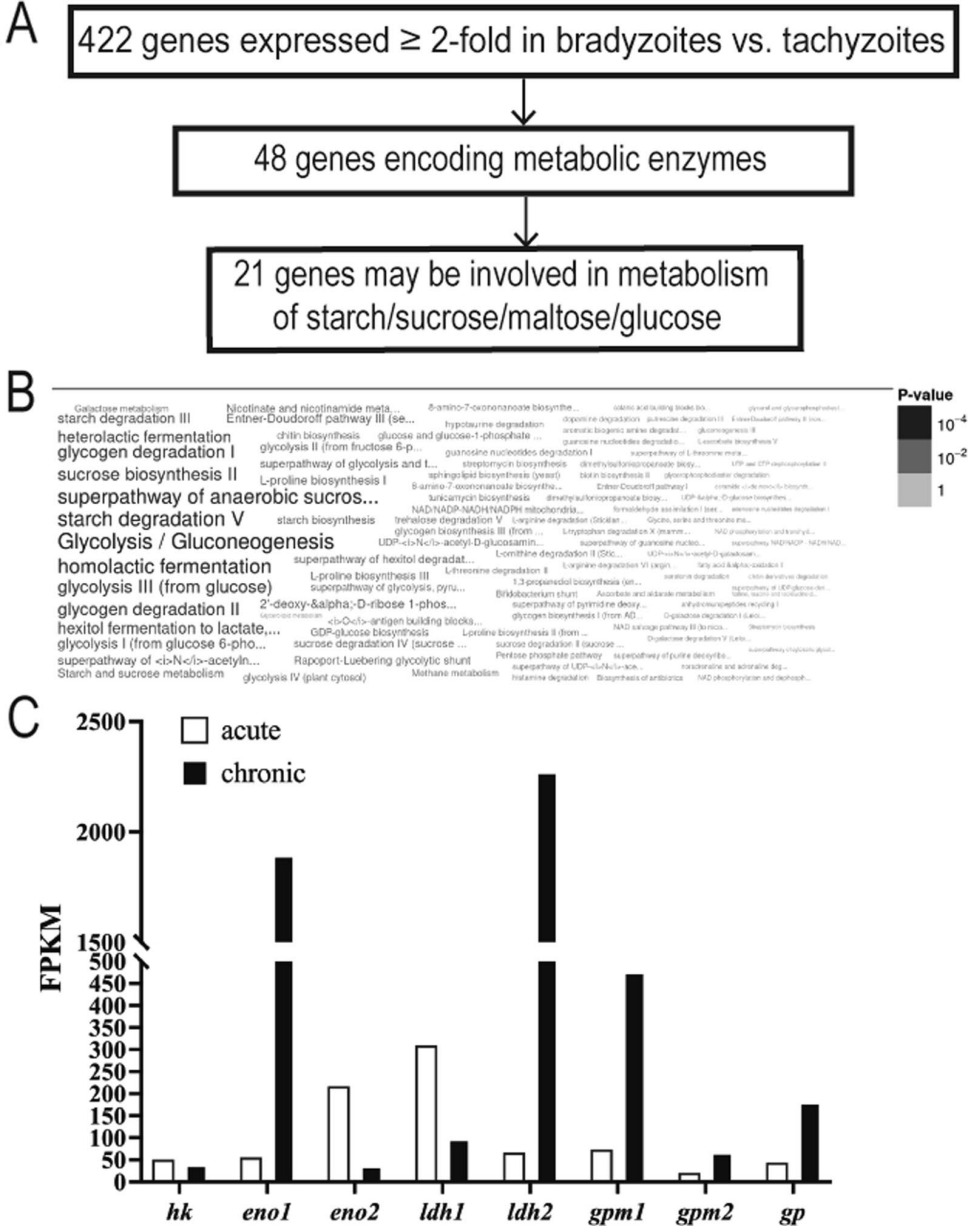
Identification of upregulated metabolic genes during *Toxoplasma* chronic infection. **A** Workflow for identification of genes associated with glycolysis and gluconeogenesis with higher expression in chronic vs. acute infection in dataset from Pittman et al*.* [12]; the analysis was performed on ToxoDB [10]. **B** Word cloud of enriched pathways among the 422 genes upregulated during chronic infection in mice. The image was generated on ToxoDB. **C** Transcript levels of differentially regulated glycolytic and gluconeogenic enzymes in *Toxoplasma*. Values were obtained from Pittman et al*.* dataset available on ToxoDB version 54

Comparative transcriptomic and proteomic analyses [22] revealed that *Toxoplasma* expresses stage-specific proteins which enable the parasite to survive and to be efficiently transmitted between hosts. We mined the transcriptional data from Pittman et al*.* [12] available on the commonly used *Toxoplasma* Informatics Resources database (ToxoDB) [10, 23] to specifically identify metabolic enzymes involved in gluconeogenesis and glycolysis that are significantly upregulated at least 2 folds in chronic vs. acute infection. Of the 422 genes upregulated in chronic infection, our analysis revealed 21 that are specifically associated with carbohydrate metabolism (Fig. 1A, B, Additional file 1). As expected, these genes include well-known glycolytic isoenzymes involved in tissue cyst formation, such as lactate dehydrogenase 2 (*ldh2*) [24] and enolase 1 (*eno1*) [25]. Interestingly, unlike *ldh1/ldh2* and *eno1/eno2* which are expressed in a stage-dependent manner, both PGM isoforms (*pgm1* and *pgm2)* were upregulated 6.4 and 3.1 folds, respectively, in the chronic stage, 28 days post-infection (dpi) [12]. Transcriptional analyses of gene expression at 28, 90, and 120 dpi from Garfoot et al. [26] indicate that unlike *pgm2* whose expression remained similar up to 120 dpi, *pgm1* transcripts further increased from 28 to 120 dpi. Together, this analysis strongly suggests that transcriptional regulation of *pgm1/pgm2* may be critical for the development and/or maintenance of tissue cysts in mice. Furthermore, the increased expression of PGM1 during chronic infection and its enzymatic activity at the intersection of energy storage and production pathways, namely glycolysis and amylopectin metabolism, warrant determining the role of this enzyme during *Toxoplasma* growth and differentiation.

### Disruption of *pgm1* does not hinder parasite growth in vitro

**Fig. 2:**
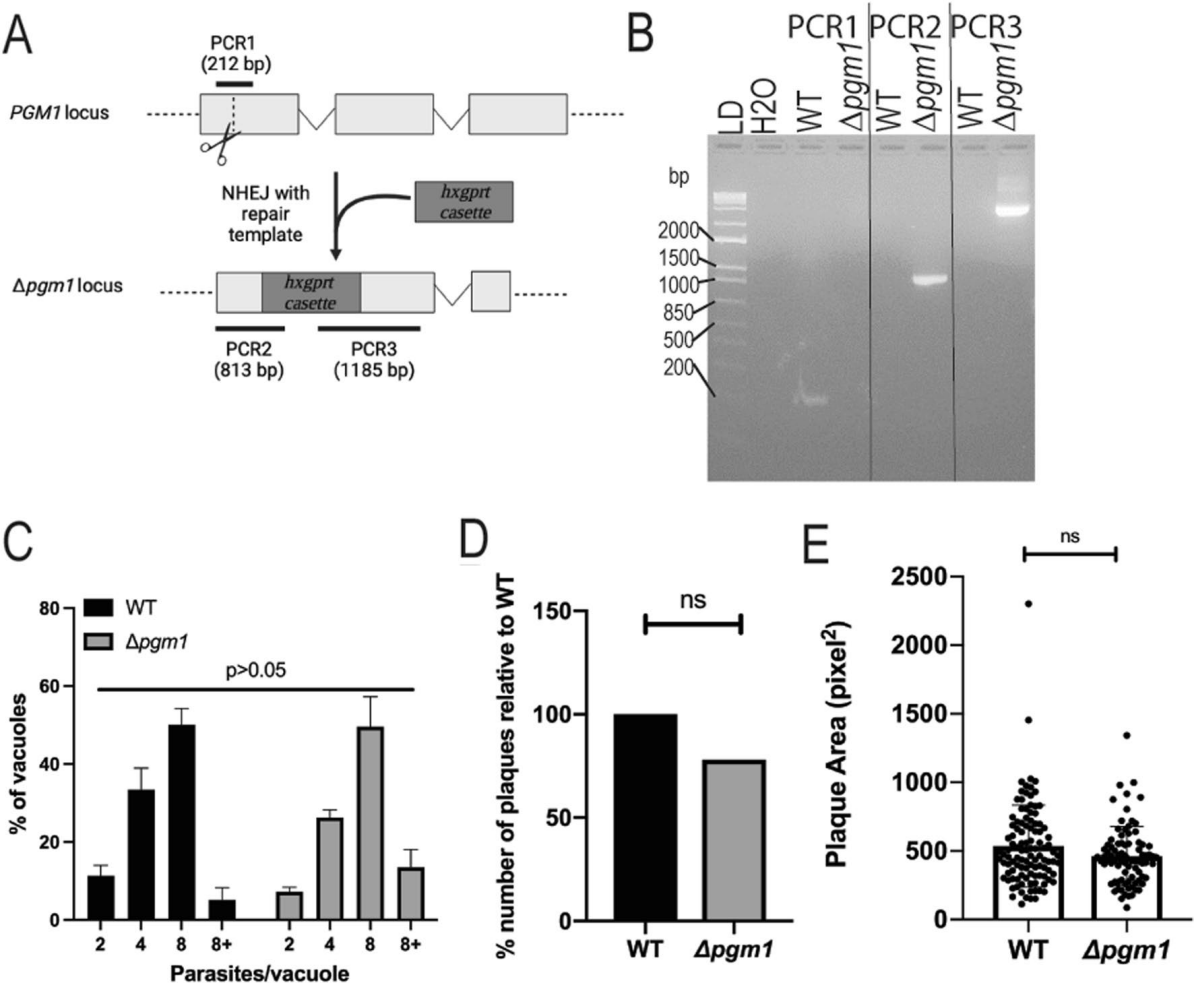
Disruption of *pgm1* and growth assays. **A** Schematic representation of disruption of *pgm1* using CRISPR-Cas9 gene-editing system for nonhomologous insertion of the *hxgprt* selectable marker cassette. The dotted line represents the region in the first exon of *pgm1* targeted by the small guide RNA (sgPGM1). **B** Image of DNA gel electrophoresis of PCR1-3 performed using DNA from wildtype (WT) and mutant (*Δpgm1*) to demonstrate integration of the *hxgprt* expression cassette at the *pgm1* locus. The expected product for PCR1 (212 bp) was obtained only for WT while products for PCR2 (813 bp) and PCR3 (1185 bp) were amplified only with *Δpgm1* DNA. **C** Intracellular growth. HFFs were infected with 1.2 × 10^5^ WT or *Δpgm1* parasites for 24 h in cDMEM. Monolayers were fixed and stained with antibodies raised against SAG1 (tachyzoite surface marker) and GRA7 (PV marker). Intracellular parasites were enumerated in at least 20 vacuoles/strain/experiment, N = 3 independent experiments; error bars = standard error of the mean; *p*-value was determined by Chi-square test. **D** Total numbers of plaques counted 10 days after infection of HFFs with 250 WT or *Δpgm1* parasites. **E** Plaque areas were determined for 85 WT and 109 *Δpgm1* plaques using Fiji/ImageJ in pixels^2^, N = 3 replicates/strain in a single experiment, error bar = standard deviation; ns: *p*-value > 0.05 by nonparametric Mann–Whitney test

To determine the contribution of PGM1 to *Toxoplasma* growth, we used the CRISPR-Cas9 gene-editing system to create an insertional mutant Me49*ΔhxgprtΔpgm1 (Δpgm1)* by introducing a *hxgprt* selection cassette at the *pgm1* locus [16] (Fig. 2A, B, Additional file 1: Figure S1). We assessed the intracellular growth of *Δpgm1* parasites vs. WT 24 h after infection of HFFs in glucose replete growth medium. SAG1-positive parasites inside GRA7-positive vacuoles were enumerated. We found similar numbers of *Δpgm1* vacuoles with either 2, 4, or ≥ 8 parasites as WT (Fig. 2C). Likewise, no significant differences in plaque numbers and sizes were observed 10 days after infection (Fig. 2 D, E). Thus, as previously reported for *Toxoplasma* RH strain [15, 27], our data indicate that PGM1 is dispensable for *Toxoplasma* intracellular growth and lytic cycle in vitro, albeit in glucose-rich conditions.

### *pgm1-*defective parasites produced smaller amylopectin-containing cysts in vitro

**Fig. 3:**
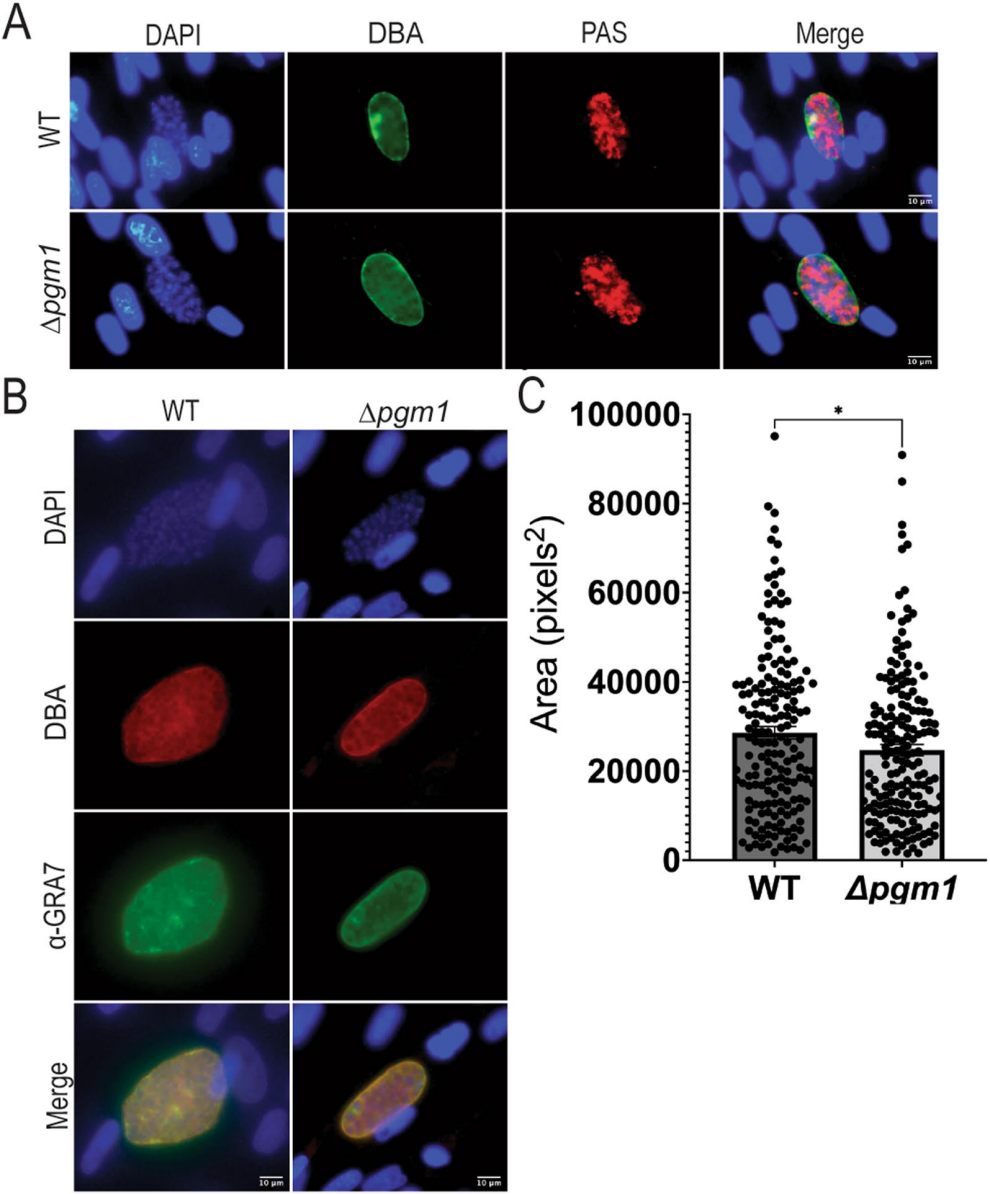
In vitro stage conversion assay. **A** Representative fluorescence images of amylopectin-containing WT and *Δpgm1* cysts at 4 days post-induction. Infected monolayers were stained with PAS to detect amylopectin (red), DBA to label the cyst wall (green), and DAPI for nuclei (blue); Scale bar = 10 microns. **B** Representative images of WT and *Δpgm1* cysts 4 days post-induction in vitro. The images are representative of the mean value of cyst areas for each strain. Cysts were stained with DBA (red), anti-GRA7 (green), and DAPI (blue); Scale bar = 10 microns. **C** Quantification of cyst sizes. The areas of 176 WT and 185 *Δpgm1* cysts were determined in pixels^2^ at 4 days post-induction from 3 independent experiments; *p = 0.0362 by nonparametric Mann–Whitney test

Given the upregulation of *pgm1* in chronic infection, we tested whether disruption of *pgm1* would impede tissue cyst formation. We induced tachyzoites to differentiate into bradyzoites in nutrient-poor, alkaline conditions in ambient CO_2_ [21]. After 4 days, we stained the monolayers with *Dolichos biflorus* agglutinin (DBA) to detect the cyst wall and Periodic Acid Schiff (PAS) to visualize amylopectin [8]. Both WT and *Δpgm1* parasites produced PAS-positive cysts (Fig. 3A), suggesting that PGM1 is not essential for amylopectin accumulation during stage conversion in vitro. However, further studies are required to determine any differences in the relative amount of this polysaccharide between WT and *Δpgm1* cysts*.* Interestingly, *Δpgm1* cysts were on average ~ 4060 pixels^2^ smaller than WT (*p* = 0.0362 by Mann–Whitney test, Fig. 3C). Together, our results indicate that although PGM1 is not required for stage conversion and amylopectin storage, the enzyme contributes to optimal cyst development in vitro.

## Discussion

PGM1 is one of two PGM isoforms differentially expressed in *Toxoplasma* [10, 12, 28]. In this study, we showed that disruption of *pgm1* in a cyst-forming *Toxoplasma* strain did not prevent intracellular growth or completion of the lytic cycle in glucose-replete conditions, corroborating previous studies in non-cyst forming Type I tachyzoites [15, 27]. Our observation that tachyzoites lacking *pgm1* could differentiate into bradyzoites in the absence of glucose further supports the nonessential role of PGM1 and PGM1-dependent glucose-6-phosphate production in tachyzoites as suggested by Imada et al. [29]. Interestingly, PGM1 has been implicated in Ca^2+^-dependent microneme secretion in tachyzoites [11, 13, 15], and thus, like functionally characterized PGMs in other organisms [9, 30], it may play an unconventional role during *Toxoplasma* development.

Additionally, the absence of *pgm1* did not abrogate amylopectin biosynthesis and storage, probably due to functional compensation with PGM2. While both *pgm1* and *pgm2* transcripts are higher in bradyzoites than tachyzoites [28], the proteins share only 25% homology. PGM2 has a significantly lower enzymatic activity than PGM1 [29]. Interestingly, Saha et al*.* [15] demonstrated that PGM2 didn’t compensate for the deletion of PGM1 in the context of Ca^2+^-regulated microneme secretion in tachyzoites.

Although glycolysis is not required for tachyzoite viability, it is critical for tissue cyst formation and pathogenesis in mice [31]. Parasites lacking hexokinase, the first enzyme in glycolysis that catalyzes the phosphorylation of glucose to glucose-6-phosphate, produce smaller cysts in vitro [31]. This phenotype was recapitulated in *pgm1*-defective parasites, further supporting the importance of glycolytic intermediates during cystogenesis. While the bradyzoite burden of PGM1-deficient cysts and their infectivity remain to be determined, it is plausible that the parasites inside these mutant cysts have decreased resistance to proteases and are less infectious following oral infection, as previously shown for Bradyzoite Pseudokinase 1 (BPK1) mutants [32]. Because the absence of PGM1 does not significantly alter the replication rate of tachyzoites, it is conceivable that the bradyzoite burdens in the mutant and wildtype cysts be comparable. This assertion is supported by Watts et al. who showed that cyst size is not a strong predictor bradyzoite burden [33].

Overall, this study suggests that PGM1 is not critical for *Toxoplasma* growth and differentiation; however, it is required for optimal cyst maturation, which is critical for the establishment of chronic *Toxoplasma* infections. Future studies are needed to parse out the interplay and diverse activities of *Toxoplasma* PGMs and understand how they affect central carbon metabolism and developmental differentiation in this ubiquitous parasite. PGMs are among several metabolic enzymes whose transcripts are significantly upregulated during chronic infection with *Toxoplasma*. While PGM1 was our initial focus, future work will evaluate the contributions of other poorly characterized glycolytic enzymes identified in our bioinformatic search. Similar to PGM1, these enzymes may be critical to *Toxoplasma* biology and serve as potential therapeutic targets against chronic toxoplasmosis.

## Limitations

Due to institutional infrastructure failures that resulted in the loss of all parasite lines, including the ones used here, we were unable to perform complementation studies or growth assays in the presence or absence of various carbon sources. We also did not quantify PAS staining to identify any difference in amylopectin accumulation between WT and mutant parasites.

## Supplementary Information

**Additional file 1:** List of primers used in this study and list of 21 genes associated with glycolysis and gluconeogenesis with higher expression in chronic vs. acute infection in mice. Data was obtained from Pittman *et al*. dataset available on ToxoDB.

## Data Availability

The transcriptional dataset used in this study is publicly available on ToxoDB version 54 (www.toxodb.org). The authors declare that all data generated supporting the findings of this study are available within the article and its Additional file 1. Plasmids, parasite and host cell strains (except for the mutant strain) are available from the corresponding author upon request.

## Abbreviations

BSA: Bovine serum albumin
DBA: *Dolichos biflorus* agglutinin
DHFR: Dihydrofolate reductase
HFF: Human foreskin fibroblast
HXGPRT: Hypoxanthine-xanthine-guanine phosphoribosyltransferase
PAS: Periodic acid schiff
PBS: Phosphate-buffered saline
PCR: Polymerase chain reaction
PGM: Phosphoglucomutase
PV: Parasitophorous vacuole
RT: Room temperature
WT: Wildtype strain
