# Supplementary material for: Phosphoglucomutase 1 contributes to optimal cyst development in Toxoplasma gondii
Source: BMC Res Notes. 2022 May 21;15:188. doi: 10.1186/s13104-022-06073-5 (PMC9123713; doi:10.1186/s13104-022-06073-5)

**Table S1: List of primers used in this study**

| **Primer sequence** | **Description** |
| --- | --- |
| caatctctcgctgcaaccatgttttagagctagaaatagc | Forward primer to replace sgUPRT with sgGPM1 in pSAG1::Cas9-U6::sgUPRT |
| aacttgacatccccatttac | Reverse primer to replace sgUPRT with sgGPM1 in pSAG1::Cas9-U6::sgUPRT |
| gcgctgcaggacgcagcgagcggttttaacctttctcttgatcagcacgaaaccttgca | Forward primer to amplify pDHFR-*hxgprt* from pGRA1 (gift from the Boothroyd lab) |
| ctgggatccccttgacgcaagacacatttcctgcttctcgtcgcggcttatttagttaa | Reverse primer to amplify pDHFR-*hxgprt* from pGRA1 |
| ctcgtctctgtctgtgttttgc | PCR 1/2, Forward primer in *gpm1*, sequencing primer |
| cgaatcacacgtttcaacactt | PCR 1/3, Reverse primer in *gpm1*, sequencing primer |
| aactctgtccttgaccaatcc | PCR 2, Reverse primer to verify for 5’ integration, sequencing primer |
| ctttcttgtgcccccccactg | PCR 3, Forward primer to verify for 3’ integration, sequencing primer |

**Table S2: List of 21 glycolytic and gluconeogenic genes upregulated during chronic infection**

| **Gene ID** | **Description** | **Fold change** |
| --- | --- | --- |
| TGME49_297060 | phosphoglycerate mutase PGMII | 2.25 |
| TGME49_257990 | heat shock protein 101, putative | 2.59 |
| TGME49_240890 | 6-phosphofructokinase | 2.79 |
| TGME49_281400 | phosphofructokinase domain-containing protein | 2.96 |
| TGME49_216810 | 5'-nucleotidase, C-terminal domain-containing protein | 3.07 |
| TGME49_318580 | glucosephosphate-mutase GPM2 | 3.09 |
| TGME49_316520 | 1,4-alpha-glucan-branching enzyme | 3.2 |
| TGME49_264000 | aldehyde dehydrogenase | 3.94 |
| TGME49_226910 | Amylo-alpha-1,6-glucosidase | 3.95 |
| TGME49_310670 | glycogen phosphorylase 1, putative | 4.15 |
| TGME49_236070 | pyrroline-5-carboxylate reductase | 4.99 |
| TGME49_283780 | glucose-6-phosphate isomerase GPI | 5.95 |
| TGME49_239620 | 5'-nucleotidase, C-terminal domain-containing protein | 6.06 |
| TGME49_285980 | glucosephosphate-mutase GPM1 | 7.71 |
| TGME49_256760 | pyruvate kinase PyK1 | 7.81 |
| TGME49_222160 | aldehyde dehydrogenase | 12.87 |
| TGME49_202300 | inosine triphosphate pyrophosphatase, putative | 13.46 |
| TGME49_309930 | melibiase subfamily protein | 16.78 |
| TGME49_291040 | lactate dehydrogenase LDH2 | 31.46 |
| TGME49_268860 | enolase 1 | 38.86 |
| TGME49_238200 | alpha/beta hydrolase fold domain-containing protein | 49.37 |

Fig. S1: Full blot of Fig. 2B


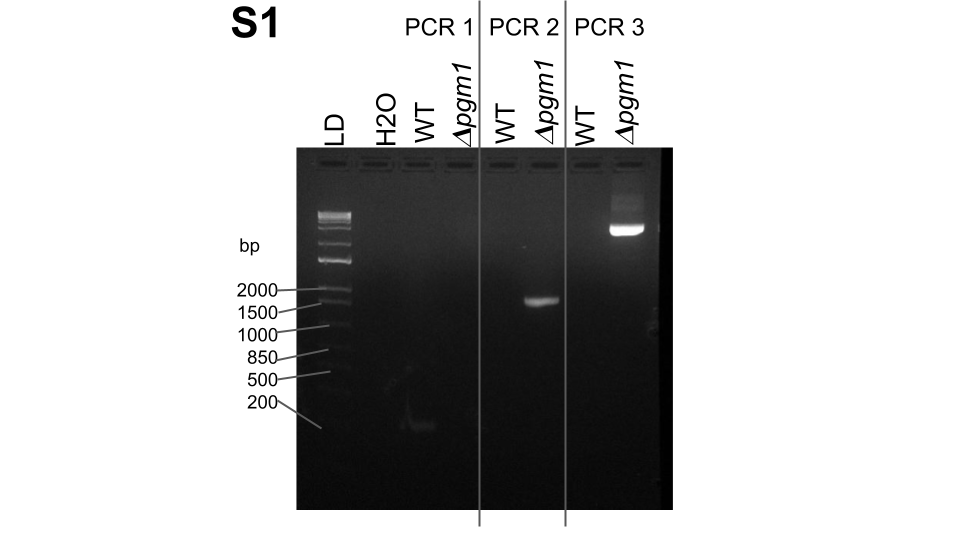

Supplement: Supplementary file 1 — Additional file 1: List of primers used in this study and list of 21 genes associated with glycolysis and gluconeogenesis with higher expression in chronic vs. acute infection in mice. Data was obtained from Pittman et al. dataset available on ToxoDB. [file 13104_2022_6073_MOESM1_ESM.docx]
